# Spontaneous Hepatic Rupture in the Setting of Peliosis Hepatis and End-Stage Renal Disease

**DOI:** 10.7759/cureus.49567

**Published:** 2023-11-28

**Authors:** Muhammad Abdullah, Manahil Rashid, Faryal Shoaib

**Affiliations:** 1 Department of Internal Medicine, Shifa Tameer-e-Millat University, Shifa College of Medicine, Islamabad, PAK; 2 Department of Internal Medicine, Shifa International Hospital Islamabad, Islamabad, PAK

**Keywords:** end-stage renal disease, spontaneous hepatic rupture, hemodialysis, blood sepsis, peliosis hepatis, hemoperitoneum

## Abstract

Rare and sometimes fatal, spontaneous hepatic rupture (SHR) is frequently documented in conjunction with various benign and malignant hepatic tumors, peliosis hepatis (PH), amyloidosis, and polyarteritis nodosa. PH is a rare disease characterized by the presence of sinusoidal dilation and blood-filled cysts throughout the hepatic parenchyma. Handling and identifying this condition can be challenging, particularly in the absence of a history of liver cirrhosis or a tumor. The present case involves a 61-year-old male with a SHR and PH, accompanied by a significant history of end-stage renal disease (ESRD) over the past year. The patient presented to the emergency department with a three-week history of right flank pain. Hemoglobin levels were found to be low; the Glasgow Coma Scale (GCS) score was progressively decreasing. A computed tomography (CT) scan revealed a rupture of the right liver capsule, hemoperitoneum, PH, and an edematous gall bladder. The current case illustrates the diagnosis and management of PH and hemoperitoneum.

This case emphasizes the challenging diagnosis of this potentially fatal liver complication in an outwardly healthy male, highlighting the connection between PH and ESRD.

## Introduction

The rare disease peliosis hepatis (PH) is characterized by sinusoidal dilatation and blood-filled cysts throughout the hepatic parenchyma. The majority of diagnosed PH patients have no symptoms; thus, it is frequently discovered by chance as a hypervascular tumor on computed tomography (CT) or magnetic resonance imaging (MRI) [1]. One of the major complications arising from PH is spontaneous hepatic rupture (SHR), with its earliest description dating back to 1844 by Abercrombie J. [2]. It still remains a rare ailment with a high fatality rate and no standardized treatment [1]. The circumstances result from a breach of Glisson’s capsule due to intra-parenchymal hemorrhage leading to SHR. Poorly supported or weak tissue exposed to a physiological event, such as a change in systolic blood pressure, might lead to SHR [3]. Another theory for SHR proposes that the growth of tumors, whether malignant or benign, is linked to progressive parenchymal venous blockage brought on by tissue pressure, the formation of thrombus, or vascular invasion [4].

We present a case involving a 61-year-old male on hemodialysis with a history of hypertension and end-stage renal disease (ESRD), who experienced a SHR with PH leading to a life-threatening hemorrhage. In the context of patients undergoing hemodialysis, liver diseases have been documented in association with exposure to 'vinyl chloride' and refractile particles of silicone [5]. This case report aims to delve into the available data and potential hypotheses, providing insights into the intricate association between ESRD treatment and the occurrence of PH.

This case report was previously presented as a poster abstract at the 2023 Shifa Annual Scholars Day on August 29, 2023.

## Case presentation

A 61-year-old man presented to the emergency department of our hospital with acute abdominal pain and abdominal distension. He has been undergoing hemodialysis three times a week for ESRD for nearly one and a half years. The patient also has a past history of hypertension spanning five years, for which he was currently receiving carvedilol (a combined alpha and beta-blocker) and amlodipine (a calcium blocker), excluding steroids and immunosuppressants. The only radiological examination conducted prior to treatment initiation was an abdominal ultrasound, which did not reveal any tumor or signs of chronic liver disease or portal hypertension. A few days prior to admission, the patient received a prescription for cefixime and hydrocortisone from a different physician to address a bacterial infection and widespread inflammation. Upon admission, these two drugs were discontinued and the physical examination indicated an unconscious patient with a Glasgow Coma Scale (GCS) score of 9/15, hemodynamically and respiratory unstable, icteric, and with a distended abdomen, with lower extremity skin blisters and vasculitis. There were no apparent signs of portal hypertension or hepatocellular insufficiency.

Laboratory parameters at the presentation are mentioned in Table 1which showed low hemoglobin, predominantly direct hyperbilirubinemia, associated with cytolysis and hepatic cholestasis. Serum aspartate aminotransferase (AST), alanine aminotransferase (ALT), and alkaline phosphatase (ALP) were markedly elevated. Elevated inflammatory markers were observed, with significantly increased white blood cell (WBC) count and C-reactive protein. Procalcitonin levels were notably high at 2.5 ng/ml, normal value is (≤ 0.10ng/ml). Renal function remained preserved, and the blood ionogram exhibited no abnormalities. The ascites puncture revealed macroscopically hematic fluid.

**Table 1:** Patient's initial and repeat lab investigations along with respective normal values ALT: Alanine transaminase, AST: aspartate transaminase, ALP: alkaline phosphatase, WBC: white blood cell

Lab Investigations	Initial Lab Findings (On Admission)	Repeat Lab Findings (After 10 Days)	Normal Values (in males)
Hemoglobin (g/dL)	6	12	13.0 – 18.0
WBC (UL)	20000	8900	4000 – 11000
C-Reactive Protein (mg/L)	236.0	61.4	≤ 5.0
AST (U/L)	4573	306	≤ 50.0
ALT (U/L)	398	120	≤ 50.0
ALP (IU/L)	505	485	40 – 130
Total Bilirubin (mg/dL)	2.40	1.75	≤ 1.2
Direct Bilirubin (mg/dL)	1.60	1.21	≤ 0.30

**Figure 1:**
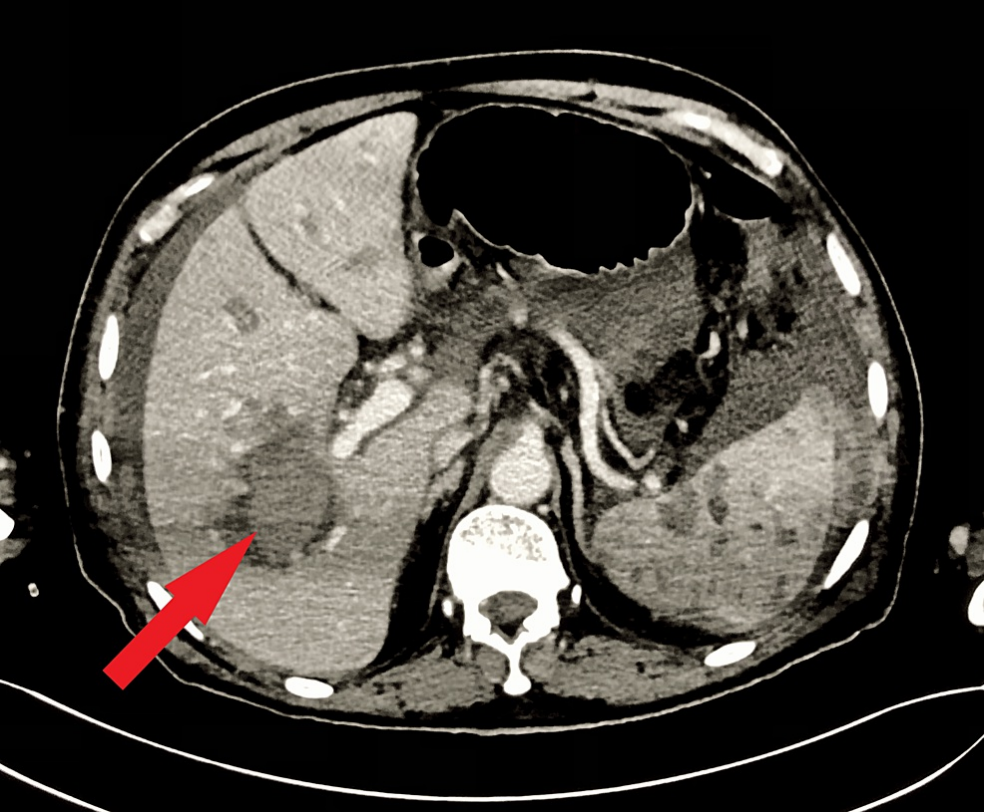
Axial computed tomography (CT) scan of the abdomen revealing peliosis hepatis (arrow)

A dynamic CT scan of the liver was conducted, revealing a significant intrahepatic hematoma in the right lobe. The imaging also identified hypodensities in both the splenic and hepatic regions, along with hepatic sinusoidal dilations, leading to the diagnosis of PH (Figure 1). Additional notable findings encompassed shrunken and scarred kidneys, bilateral pleural effusion, and layering sludge along the dependent part of the gall bladder. He was later admitted for ongoing abdominal discomfort, continuous monitoring, sequential liver function tests (LFTs), and consultation with a gastrointestinal specialist.

**Figure 2:**
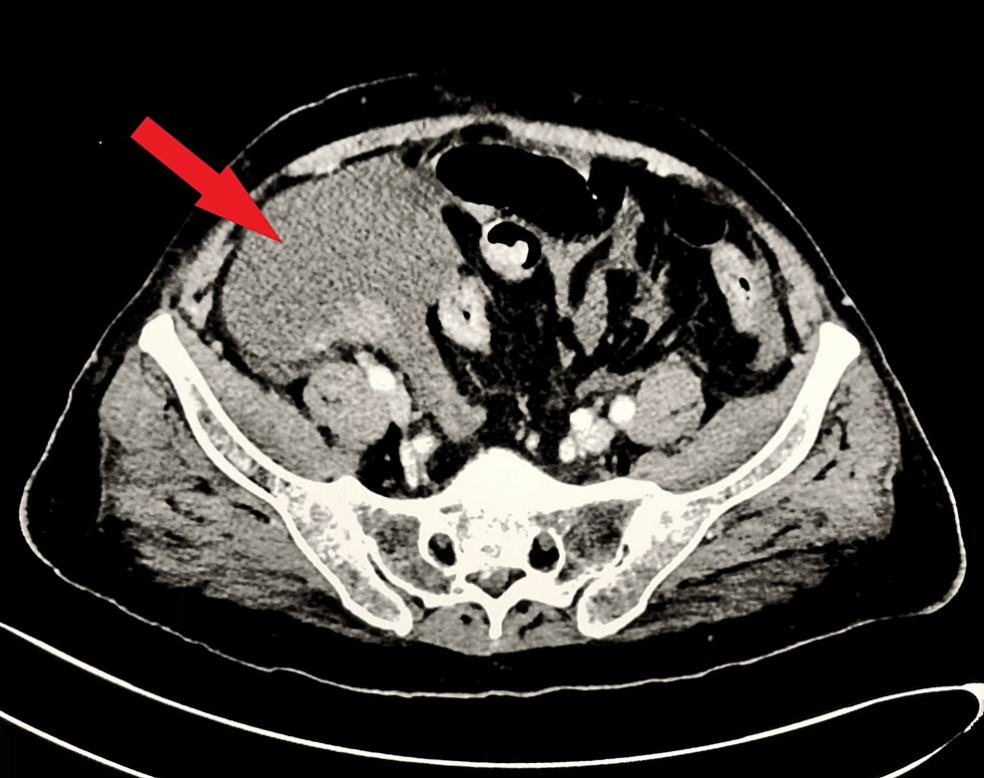
Axial computed tomography (CT) scan of the pelvic region revealing hemoperitoneum (arrow)

During the patient's hospital stay, there was a spontaneous worsening of abdominal pain, accompanied by a blood pressure of 95/52 mmHg and tachycardia. The CT scan revealed acute hepatic rupture with hemoperitoneum, and hemorrhage extended into the pelvis (Figure 2). On the second day of the patient's hospitalization, the General Surgery team was consulted, resulting in the successful resuscitation of the patient facilitated by an immediate transfusion of packed red blood cells. Subsequently, the patient was transferred to the intensive care unit. By the third day of hospitalization, an infectious disease specialist was consulted, suggesting the patient was developing blood sepsis and a septic hematoma secondary to a lower respiratory tract infection. Antibiotics (meropenem and vancomycin) were prescribed intravenously. Due to concerns about skin blisters, the dermatology department recommended a skin biopsy, revealing benign ulcers with no granulomatous inflammations or malignancy. Topical Mycitracin ointment was administered. By the 10th day of hospitalization, repeat lab investigations revealed (Table 1) reduced WBC and increased hemoglobin levels. Repeat LFTs showed progressively decreasing ALT, AST, ALP, total bilirubin, and direct bilirubin levels. The blood CRP had dropped. A positive β-d-Glucan test, indicative of invasive fungal infections, confirmed septic encephalopathy, elucidating the reason for the low GCS score. The previously prescribed antibiotics were discontinued, and fluconazole was initiated.

On the 14th day of hospitalization, the patient recovered and was discharged without the need for emergent surgical intervention, in line with general surgery recommendations. The patient's requirement for right lobe embolization to control recurrent bleeding was reviewed, but it was decided against due to the associated dangers and side effects. These potential complications included non-target embolization, liver infection leading to abscess formation, and ultimately liver decompensation. Given the limited therapeutic options considering the patient's condition, a liver transplant was considered, though it is not an established treatment for hepatic rupture. The decision was made to observe the patient's PH. A follow-up CT scan conducted three months after discharge revealed no growth of PH.

## Discussion

PH is an exceedingly rare condition typically brought to the surgeon's attention following a spontaneous rupture of the lesion, often resulting in significant hemoperitoneum [6]. A recent analysis by Srinivasa et al. highlighted that a majority of PH patients present with bleeding, which stands as the primary cause of spontaneous hepatic hemorrhage and liver hematoma. Various factors contribute to this condition, including a spectrum of benign and malignant hepatic tumors, amyloidosis, polyarteritis nodosa, HELLP syndrome, and acute fatty liver during pregnancy [3]. The etiology of PH has been associated with factors such as alcohol consumption or the use of immunosuppressive medications like cyclosporine, azathioprine, oral contraceptives, and anabolic steroids [7]. In our case, the intravenous administration of hydrocortisone before admission to our hospital might have potentially exacerbated the existing condition, leading to its discontinuation upon admission. It is worth noting that none of the aforementioned etiologies applied to our specific case.

Patients with ESRD undergoing hemodialysis have shown a positive correlation with PH development. The presence of PH in these patients raises concerns regarding the potential toxicity of both the artificial kidney/dialyzer and the sterilization products used during hemodialysis, accentuating their adverse effects [8]. Degos et al. suggested that while chronic liver disease is prevalent in renal transplant recipients, its occurrence isn't an immediate consequence of transplantation. Instead, it seems associated with the hemodialysis procedure [9]. A case of PH reported in a patient with chronic glomerulonephritis depicts a 38-year-old man on hemodialysis for two years, treated with fluoxymesterone for severe anemia, leading to splenomegaly and subsequent diagnosis of PH. Remarkably, the liver appeared macroscopically normal [6]. Another case illustrated a scenario involving a hemodialysis patient experiencing unexplained abdominal pain that subsequently resolved. The patient exhibited mild hepatosplenomegaly, sterile non-malignant ascites, and elevated alkaline phosphatase levels. A liver biopsy unveiled preserved lobular architecture and patchy interstitial fibrosis, predominantly in perivenular regions, displaying characteristic features aligning with PH. These included sinusoidal dilatation and irregular spaces within the hepatic parenchyma [10]. The emergence of new cases of PH in dialysis patients prompts a reevaluation of the potential toxicity linked not only to the artificial kidney/dialyzer but also to sterilization products. This might necessitate expanding the roster of potentially harmful drugs, including nonsteroidal anti-inflammatory agents and analgesics.

Regarding the clinical presentation, typical indications of liver hemorrhage, also seen in our case, include abdominal discomfort, a general feeling of unwellness, and vomiting. The initial workup should involve an assessment of liver function, coagulation status, and tumor markers. While MRI is considered the gold standard for diagnosing PH, studies have highlighted the efficacy of dynamic CT of the liver in this diagnosis [3]. In our case, a CT scan revealed sinusoidal dilation and blood-filled cysts throughout the hepatic parenchyma, confirming PH. Histopathology, as demonstrated in a case study by Maruyama et al., proved instrumental in diagnosing PH in a patient displaying bluish-reddish-blackish liver lesions [11]. Treatment goals primarily focus on achieving durable hemostasis, often through conservative therapy or trans-arterial band embolization. For instance, in a case report by Mascagni et al., initial trans-arterial embolization followed by right hemi-hepatectomy successfully halted bleeding due to right hepatic capsule rupture and hemoperitoneum [12].

In this case, two primary concerns were identified: hemoperitoneum associated with PH and concomitant sepsis encephalopathy. Blood laboratory analysis revealed anemia and elevated levels of liver enzymes along with inflammatory markers such as white blood cell count, CRP, and Procalcitonin. Similar to findings reported by Chikamori et al. [13], the concurrent presence of hemoperitoneum and sepsis likely contributed to the patient's low GCS score upon admission. Sepsis impact extends to brain cells and macrophages, where it affects the blood-brain barrier (BBB), allowing the entry of various molecules like cytokines, lipopolysaccharide (LPS), and bacteria into the brain. The activation of mitochondria via miR370-3p, upregulated by LPS and cytokines (particularly TNF-α) through TLR-4 and TNF receptor (TNFR), induces mitochondrial injury. Additionally, molecules circulating in the blood during sepsis can hyper-activate macrophages, leading to exaggerated inflammatory responses [14]. Hence, antibiotic therapy was initiated to address the septic condition. Monitoring CRP levels throughout the antibiotic course indicated the patient's recovery from the infection. Previous literature has established a clear link between decreased erythropoietin production and anemia, further supporting the observations in our case [15].

## Conclusions

The impact of hemodialysis on PH remains uncertain due to the limited literature and inconclusive arguments available. While the occurrence of PH in ESRD patients undergoing hemodialysis is exceedingly rare, it should be considered as a potential differential diagnosis, especially when they exhibit signs such as hepatomegaly, splenomegaly, and abnormal LFTs, moreover, when liver hypodensities are detected in imaging studies, and when patients present with right flank pain alongside signs of hyperbilirubinemia.

Our clinical case, coupled with an extensive literature review, underscores the challenges associated with identifying liver complications leading to substantial hemoperitoneum in individuals without a history of hepatic illness, particularly those undergoing Hemodialysis for ESRD. This case provides valuable insights to aid medical practitioners in achieving an early and accurate diagnosis of liver complications in ESRD patients.
